# A Smartphone App to Improve Oral Anticoagulation Adherence in Patients With Atrial Fibrillation: Prospective Observational Study

**DOI:** 10.2196/30807

**Published:** 2022-01-07

**Authors:** Keitaro Senoo, Tomonori Miki, Takashi Ohkura, Hibiki Iwakoshi, Tetsuro Nishimura, Hirokazu Shiraishi, Satoshi Teramukai, Satoaki Matoba

**Affiliations:** 1 Department of Cardiac Arrhythmia Research and Innovation Graduate School of Medical Science, Kyoto Prefectural University of Medicine Kyoto Japan; 2 Department of Cardiovascular Medicine Graduate School of Medical Science, Kyoto Prefectural University of Medicine Kyoto Japan; 3 Departments of Biostatistics Graduate School of Medical Science, Kyoto Prefectural University of Medicine Kyoto Japan

**Keywords:** atrial fibrillation, smartphone app, anticoagulants, drug adherence, education, patient involvement

## Abstract

**Background:**

Poor adherence to oral anticoagulation in elderly patients with atrial fibrillation (AF) has been shown to negatively impact health care costs, morbidity, and mortality. Although various methods such as automated reminders, counseling, telephone support, and patient education have been effective in improving medication adherence, the burden on health care providers has been considerable. Recently, an attempt has been made to improve medication adherence without burdening health care providers by using smartphone apps; however, the use of the app for elderly patients with AF is still limited.

**Objective:**

The purpose of this study was to determine whether the newly developed smartphone app for patients with AF (the Smart AF), which integrates education, automatic reminder, and patient engagement strategies with a simple user interface, can improve medication adherence in elderly patients with AF.

**Methods:**

Patient enrollment was carried out by obtaining informed consent from patients with AF attending Kyoto Prefectural University of Medicine hospital between May 2019 and September 2020. Follow-up was planned at 1, 3, and 6 months after enrollment, and questionnaire reminders were automatically sent to patient apps at designated follow-up time points. A questionnaire-based survey of medication adherence was performed electronically using the self-reported 8-item Morisky Medication Adherence Scale (MMAS-8) as the survey tool.

**Results:**

A total of 136 patients with AF were enrolled in this study. During the follow-up period, 112 (82%) patients underwent follow-up at 1 month, 107 (79%) at 3 months, and 96 (71%) at 6 months. The mean age of the enrolled patients was 64.3 years (SD 9.6), and male participants accounted for 79.4% (108/136) of the study population. The mean CHADS2 (congestive heart failure, hypertension, age, diabetes, previous stroke, or transient ischemic attack) score was 1.2, with hypertension being the most common comorbidity. At the time of enrollment, 126 (93%) and 10 (7%) patients were taking direct oral anticoagulants and warfarin, respectively. For medication adherence as measured according to the MMAS-8, MMAS scores at 1 month, 3 months, and 6 months were significantly improved compared with baseline MMAS scores (all *P* values less than .01). The overall improvement in medication adherence achieved by the 6-month intervention was as follows: 77.8% (14/18) of the patients in the high adherence group (score=8) at baseline remained in the same state, 45.3% (24/53) of the patients in the medium adherence group (score=6 to <8) at baseline moved to the high adherence group, and 72% (18/25) of the patients in the low adherence group (score <6) moved to either the medium or high adherence group.

**Conclusions:**

The Smart AF app improved medication adherence among elderly patients with AF. In the realm of medication management, an approach using a mobile health technology that emphasizes education, automatic reminder, and patient engagement may be helpful.

## Introduction

Atrial fibrillation (AF), the most common chronic arrhythmia, affected 33.5 million people worldwide, in 2010, and the number is projected to double by 2030 [1,2]. AF is associated with a 5-fold increased risk of stroke, and oral anticoagulation (OAC) is required for people at moderate-to-high risk of stroke [3]. Poor adherence to medication in the real world can alter efficacy and safety estimates from randomized controlled trials, leading to poorer health outcomes and greater health care costs [4,5]. A recent meta-analysis found that suboptimal adherence to and persistence with direct oral anticoagulants (DOACs) is common [6]. For example, patients with AF do not take a DOAC every 4 days; one-third of the patients have less than 80% adherence; real-world persistence with DOACs is lower than in randomized controlled trials; and patients with poor adherence have a higher risk of stroke.

To date, various greater efforts in monitoring and interventions have been used to improve OAC adherence. For instance, the AEGEAN (Assessment of an Education and Guidance Programme for Eliquis Adherence in Non-Valvular Atrial Fibrillation) trial explored the impact of education (ie, using booklets and reminder tools) and telephone follow-up using a virtual clinic on adherence to apixaban. However, in the AEGEAN trial, electronically measured adherence did not differ between the usual care and intervention groups, with adherence rates of 88.5% and 88.3%, respectively [7]. A study by Shore et al [8] reported that enhanced pharmacist engagement and longer patient monitoring and follow-up were associated with greater adherence to dabigatran. In a study by Desteghe et al [9], remote monitoring of daily medication uses and daily remote monitoring and individualized feedback by telephone resulted in very high adherence to DOACs, with 99.0% demonstrating adherence and 96.8% demonstrating regimen adherence. FACILITA (strategies for improving dabigatran adherence for stroke prevention in patients with non-valvular atrial fibrillation) study revealed that a mixed intervention, consisting of patient education and a simple calendar reminder for drug intake, was an effective strategy to improve adherence to dabigatran (to 91% and 89% at 6 and 12 months, respectively, compared with 65% and 63% for the control group) [10]. To improve medication adherence, various methods such as reminders, counseling, telephone support, and patient education are effective; however, these long-term interventions impose a considerable burden on health care providers [11,12].

Recently, attempts have been made to increase medication adherence without burdening health care providers by using smartphone apps in various fields [13-15]. The Health Buddies app (DAE Studios) was developed as a tool to improve adherence by providing a virtual contract with the patients’ grandchildren [16]. The mAF app was developed to integrate clinical decision support, education, and patient-involvement strategies [17]. Although some success was achieved in these studies, there were problems such as the complexity of the app user interface.

The purpose of this study was to determine whether the newly developed smartphone app for patients with AF (the Smart AF app), which integrates education, automatic reminder, and patient engagement strategies with a simple user interface, can improve medication adherence in elderly patients with AF.

## Methods

### Features of the Smart AF App

**Figure 1:**
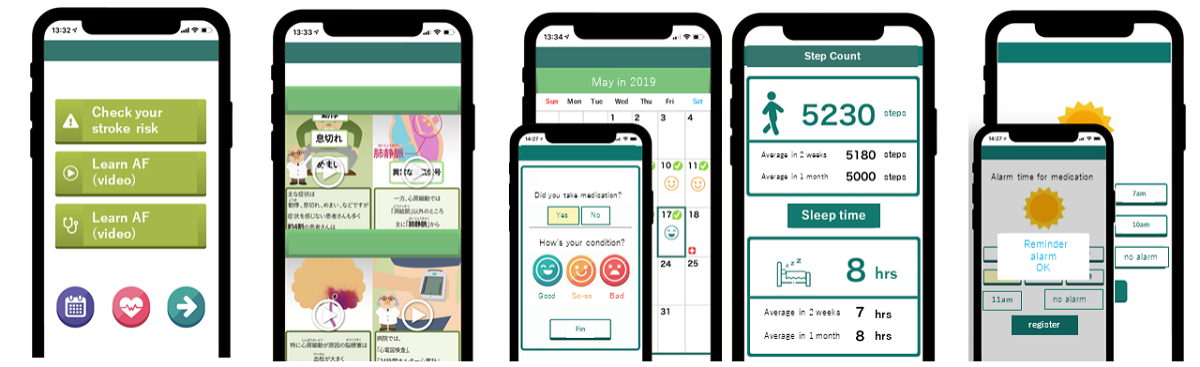
Features of the smartphone app for atrial fibrillation (the Smart AF app).

The Smart AF app was developed by Health Tech Innovation Center, in association with Kyoto Prefectural University of Medicine, funded by the BMS/Pfizer Japan Thrombosis Investigator Initiated Research Program (JRISTA). There are 3 features of the Smart AF app (Figure 1).

### Educational Program

There are 7 components to the patient educational program. With videos that are approximately 1-2 minutes in length, patients can learn about AF and learn self-management methods, including how to detect and treat AF, the importance of anticoagulation, and the treatment of comorbidities.

### Patient Engagement

After inputting information about the characteristics of their AF history, other medical history, medication information (eg, type of antithrombotic medication or other concomitant medications), and lifestyle behaviors, each patient’s CHADS2 score is automatically calculated, enabling physicians to easily understand their risk for stroke. Furthermore, the app can take inputs on step counts, sleep time, and presence or absence of symptoms and, by sharing this information with health care providers, the proactive involvement of the patient in their own care can be promoted and supported.

### Reminder Alarm

A reminder is automatically transmitted through the app in the morning and evening daily to prevent forgetting to take medication.

### Outcome Measures

Follow-up was planned at 1, 3, and 6 months after enrollment, and a reminder email for the survey was automatically sent to the patient's app at the time of follow-up. The self-reported 8-item Morisky Medication Adherence Scale (MMAS-8) was used as the survey instrument [18-20]. The MMAS-8 score assesses patients' self-reported adherence to their anticoagulant medication. According to the MMAS-8 score (range 0-8), adherence was defined as high (score 8), medium (score 6 to <8) or low (score <6).

### Eligibility Criteria for Participants

Patient enrollment was carried out by obtaining informed consent from patients with AF attending Kyoto Prefectural University of Medicine hospitals between May 2019 and September 2020. All participants provided electronic informed consent and were assigned a password. Inclusion criteria were as follows: documented diagnosis of AF; current prescription for OACs (ie, dabigatran, rivaroxaban, apixaban, edoxaban, and warfarin) for at least 3 weeks; and ownership of a mobile phone. Individuals less than 20 years of age, those with valvular AF, and those who had been taking OACs for less than 3 weeks were excluded. This single-center prospective observational study was approved by the Institutional Review Board of the Kyoto Prefectural University of Medicine (ERB-C-1429).

### Data Analysis

Statistical analyses were performed using R version 3.6.1 (R Foundation for Statistical Computing). Continuous variables are expressed as mean and SD, and categorical variables as number and percentage. MMAS-8 scores at 4 time points (baseline, 1, 3, and 6 months later) were plotted to illustrate changes in these variables over time. The Wilcoxon signed-rank test was used to evaluate differences in MMAS-8 scores between baseline and 1, 3, and 6 months. For the 96 patients who were able to complete the follow-up up to 6 months, the change in adherence from the time of enrollment to 6 months is reflected graphically by a low, middle, and high MMAS score. The percentage of patients who activated the app at least once per day is expressed as the mobile app retention rate, and the mean retention rate with SD per week at 1, 3, and 6 months was calculated and graphed. Associated factors were also identified using simple regression analysis. “Low retention,” defined as the percentage of days in which the app was activated at least once, was ≤10% during the observation period. Differences with *P*<.05 were considered to be statistically significant.

This study was funded by JRISTA. Permission for use of the MMAS-8 scale and its coding has been acquired, and a license agreement is available from MMAR, LLC, Donald E Morisky, ScD, ScM, MSPH, 294 Lindura Ct., United States.

## Results

Between May 2019 and September 2020, a total of 136 patients with AF were enrolled in this study. During the follow-up period, 112 (82%) patients underwent the follow-up survey at 1 month, 107 (79%) at 3 months, and 96 (71%) at 6 months. The mean age of the enrolled patients was 64.3 years (SD 9.6), and males accounted for 79.4% (108/136) of the study population; 89.7% (122/136) of the patients were married and had family members currently residing with them; 63.9% (87/136) of the patients had attended college as their highest level of education, and 57.3% (78/136) were currently working; 75% (102/136) were currently practicing regular dietary habits, and 19.8% (27/136) engaged in daily exercise; 74.2% (101/136) of the patients had a history of AF treatment for ≥1 year, and hypertension was the most common comorbidity, with a mean CHADS2 score of 1.2. Moreover, 38.9% (53/136) of the patients experienced palpitation symptoms, 130 (96%) patients with a Hospital Anxiety and Depression Scale (HADS)-A score of ≥8 (major anxiety), 30 (22%) patients with a HAD-D score of ≥11 (major depression), and 95 (70%) patients with a HADS-T score of ≥20 (major anxiety and depression). At enrollment, 126 (93%) patients were taking DOAC, and 10 (7%) were taking oral warfarin (Table 1).

**Figure 2:**
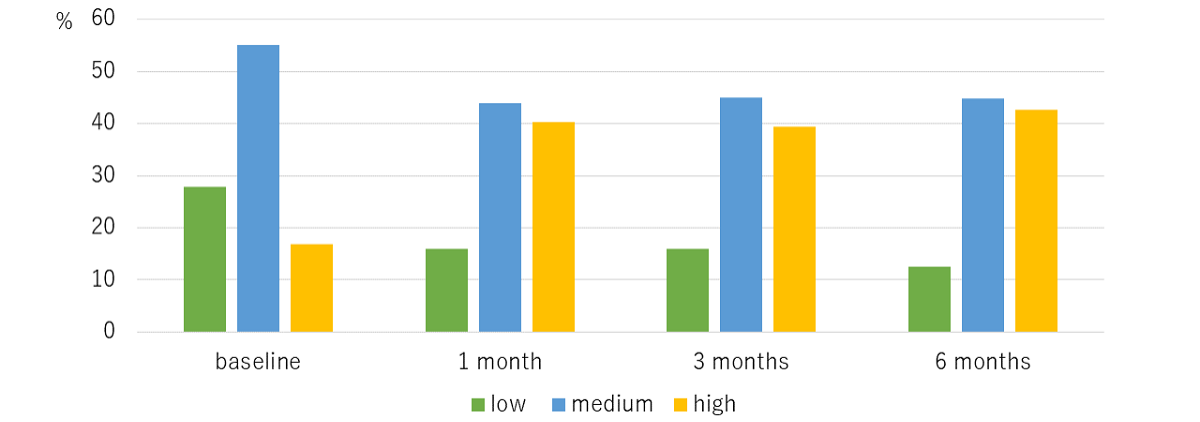
Morisky Medication Adherence Scale at baseline, 1 month, 3 months, and 6 months.

For medication adherence, as measured according to the MMAS-8, the low adherence group was 27.9% (n=38) at baseline, 16.0% (n=18) at 1 month, 15.9% (n=17) at 3 months, and 12.5% (n=12) at 6 months. Medium adherence was 55.1% (n=75) at baseline, 43.8% (n=49) at 1 month, 44.9% (n=48) at 3 months, and 44.8% (n=43) at 6 months. High adherence group was 16.9% (n=23) at baseline, 40.2% (n=45) at 1 month, 39.3% (n=42) at 3 months, and 42.7% (n=41) at 6 months, respectively. Compared with baseline MMAS scores, MMAS scores at 1 month, 3 months, and 6 months were significantly improved (all *P* values<.01) (Figure 2).

**Figure 3:**
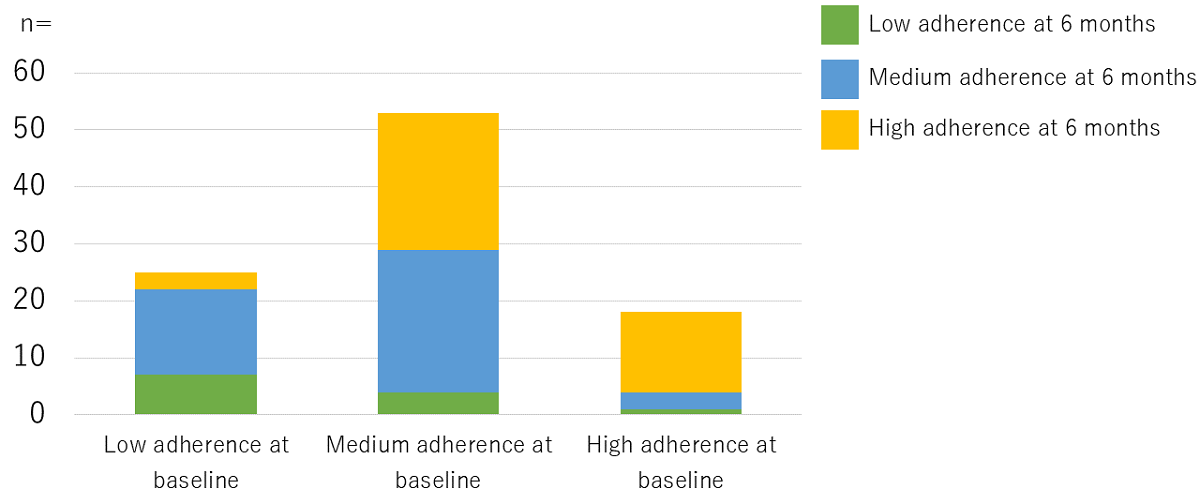
Change in adherence category from baseline to 6 months of follow-up (n=96).

Furthermore, the overall improvement in medication adherence achieved by the intervention was as follows: 77.8% (14/18) of the patients in the high adherence group at baseline remained there; 45.3% (24/53) of the patients in the medium adherence group at baseline moved to the high adherence group; and 72% (18/25) of the patients in the low adherence group moved to either the medium or high adherence groups (Figure 3).

**Figure 4:**
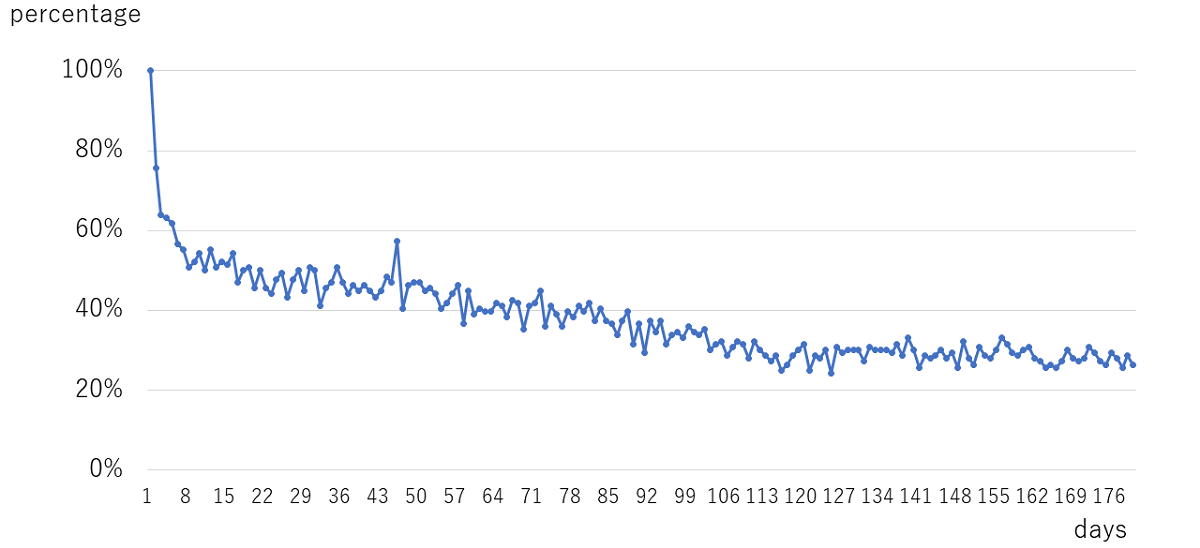
Mobile app retention.

The mobile app retention rate is a plot of the percentage of patients who activate the app at least once per day (Figure 4).

**Table 1:** Demographics of the participants (N=136).

Characteristics		Values
Age (years), mean (SD)		64.3 (9.6)
Male, n (%)		108 (79.4)
Married, n (%)		122 (89.7)
Living together, n (%)		123 (90.4)
University education level, n (%)		87 (64.0)
Full-time or part-time employed		78 (57.4)
Regular meal, n (%)		102 (75.0)
**Habit of exercise, n (%)**		
	Every day	27 (19.9)
	Sometimes	54 (39.7)
	None	55 (40.4)
Duration of atrial fibrillation (>1year)		101 (74.3)
**Type of oral anticoagulants, n (%)**		
	DOAC^a^, OD^b^ (edoxaban, rivaroxaban)	86 (63.2)
	DOAC, BID^c^ (apixaban, dabigatran)	40 (29.4)
	Warfarin	10 (7.4)
CHADS2^d^ score (SD)		1.2 (1.1)
Congestive heart failure, n (%)		18 (16.5)
Hypertension, n (%)		65 (59.6)
Age ≥75 (years), n (%)		13 (9.6)
Diabetes mellitus, n (%)		19 (17.4)
Prior stroke or TIA^e^, n (%)		9 (8.3)
With symptom, n (%)		53 (39.0)
**HADS^f^ scale, n (%)**		
	HADS-A (≥8—major anxiety; total 21 points)	130 (95.6)
	HADS-D (≥11—major depression; total 21 points)	30 (22.1)
	HADS-T (≥20—major anxiety and depression: total 42 points)	95 (69.9)

^a^DOAC: direct oral anticoagulants.

^b^OD: once a day.

^c^BID: twice a day.

^d^CHADS2: congestive heart failure, hypertension, age, diabetes, previous stroke, or transient ischemic attack.

^e^TIA: transient ischemic attack.

^f^HADS: Hospital Anxiety and Depression Scale.

Retention rates for 1, 3, and 6 months were 0.48 (SD 3), 0.36 (SD 3), and 0.27 (SD 1), respectively. In the univariate regression analysis, predictors for a higher retention rate were older age (*P*=.047), regular meals (*P*=.04), and HADS-A ≥8 (major anxiety, *P*=.04), respectively (Table 2).

**Table 2:** Predictors for retention rate.

Characteristics and variables		Values	
		B^a^	*P* value
Age		0.605	.047
Male		-0.442	.95
Married		-4.134	.67
Living together		-0.349	.97
Education level		-1.756	.77
Employed		0.096	.99
Regular meal		13.56	.04
**Habit of exercise**			
	Every day	3.023	.68
	Sometimes	3.871	.52
	None	-5.845	.33
Duration of atrial fibrillation (>1 year)		-2.087	.76
Oral anticoagulants, BID^b^ (reference as OD^c^)		1.538	.81
CHADS2^d^ score		-0.257	.90
Polypharmacy		-0.376	.98
With symptom		-1.533	.80
**HADS^e^ scale**			
	HADS-A (≥8—major anxiety)	28.875	.04
	HADS-D (≥11—major depression)	-10.946	.12
	HADS-T (≥20—major anxiety and depression)	-2.533	.69

^a^B: univariate regression analysis.

^b^BID: twice a day.

^c^OD: once a day.

^d^CHADS2: congestive heart failure, hypertension, age, diabetes, previous stroke, or transient ischemic attack.

^e^HADS: Hospital Anxiety and Depression Scale.

## Discussion

### Main Findings

The Smart AF app has been developed, which integrates patient education, reminder alarm, and patient engagement strategies without increasing the burden on health care providers. Elderly patients with AF who used the Smart AF app demonstrated significantly improved MMAS-8 at 6 months compared to baseline. The Smart AF app demonstrated a significant decrease in the low adherence group and an increase in the high adherence group at 6 months compared with baseline.

### Improvement of Adherence

Current clinical guidelines for AF advocate incorporating patient preferences regarding treatment and support and involving patients in management decisions [21,22]. The participation of patients with AF in the process of developing and refining the app through patient involvement resulted in the Smart AF app. For example, to facilitate the use of the app, age-related aspects considered to be useful in the design of mHealth (mobile health) tools, including large screens, large fonts, and ease of navigation, were incorporated into it. As a result, we adopted a simple layout and large navigation buttons that are easy to use, even by elderly individuals. Among the few studies that evaluated the effectiveness of smartphone apps in improving medication adherence in those with AF, research investigating the mAF app revealed that it had a complex design with a great deal of content, such as education programs, clinical decision support material, patient involvement in self-care, structured follow-up, and many areas that users needed to manipulate manually [17]. Regarding the Health Buddies App, there are some problems, such as a lack of interest in the characteristic games of the app, to the point that grandchildren who become “Buddies” must also participate; moreover, although achieving tasks and goals inside the app was fun, long-term behavioral change was difficult because these achievements do not correlate directly with patient health conditions [16].

The Smart AF app was developed with a focus on patient education, reminder alarms, and patient engagement in self-care, and we tried to keep the app simple without any other features. In fact, the FACILITA study also revealed that a mixed intervention, consisting of patient education and a simple calendar reminder of drug intake, was an effective strategy to improve adherence to dabigatran (to 91% and 89% at 6 and 12 months, respectively, compared with 65% and 63% for the control group) [10]. Therefore, despite the simplicity of the app’s contents, we believe that not only did the reminder alarms encourage patients to take their daily medication, but by having them record their medication in the app's calendar, we could encourage their participation and make them more aware of their engagement in self-care by managing their daily health.

Previous studies have also reported that tailor-made educational interventions can significantly improve anticoagulation management of warfarin [23]. In addition, the IMPACT-AF (Integrated Management Program Advancing Community Treatment of Atrial Fibrillation) study, which investigated the use of oral anticoagulation in patients with AF, found that it was improved by a multifaceted, multilevel (including at medication initiation as well as at follow-up) educational program implemented by physicians [24]. The Smart AF app has educational contents to help patients quickly solve their questions about AF. Thus, the smart AF app can enhance patient education and medication reminders at any time and place. In addition, we believe that we have succeeded in encouraging long-term patient engagement in self-care by avoiding complex operations and time-consuming input as much as possible.

### Retention Rate of the Smart App

The decrease in the retention rate of the app over time is an important issue to be considered. The mean retention rates at 1, 3, and 6 months were 0.48 (SD 3), 0.36 (SD 3), and 0.27 (SD 1), respectively. A decline in app use over time is also a concern that has been highlighted in previous reports [25].

Although the app offers the advantage of completely remote recruitment and enrollment, lack of human communication may mean less motivation for the participants to continue compared with studies conducted face-to-face.

The Smart AF app was designed to pop up notifications (reminder) every morning and evening, even when the app had not been activated or opened. Such pop-up feature may have succeeded in improving medication adherence regardless of whether the app was activated or not. However, long-term use is essential for the app to affect users. Clearly, additional efforts to improve retention rates are necessary. The characteristics associated with the retention time of apps have not been well studied. In a study of patients with asthma, being female and older was related to longer retention [26]. In our study, 95 patients enrolled in the study had an HADS-T score of 20 or higher (major anxiety and depression), and most of them had anxiety and depression. As shown in Table 2, older patients, patients with a regular diet, and patients with anxiety had higher retention rates. Conversely, younger patients, those with irregular diets, and those without anxiety had lower retention rates. To deliver mHealth effectively, it is important to identify patient domain factors, such as psychological factors, dietary regularity, and age, that mark suitable candidates for the app.

In the future, to further increase continued app usage rates, we believe that we must work to incorporate an interactive design; more specifically, design elements that respond immediately to patient operations and behaviors.

### Limitations

There were limitations to our study and findings. First, this was a single-center study, and the patients were not prospectively randomized into intervention (the app group) and usual care groups. Second, while important in this type of study, the reliance on measurement of self-reported results is a challenge often encountered. The data obtained from these indices may be supported by more objective observations. For example, we used a self-reported medication adherence tool to examine medication adherence. Older adults taking multiple medications may not be able to accurately report their medication use status due to poor memory or confusion. Therefore, it may be useful to complement self-reported measures using more objective measures of medication adherence (eg, medical record review and pharmacy documentation). Third, other new interventions, strategies, and technologies designed to enhance long-term adherence to DOACs need to be developed and investigated because patients with AF are a large and diverse patient population, and not all will have access to newer mHealth tools. Nonadherence is often caused by a multitude of factors, indicating the necessity of providing patients with tailored and more personalized tools. Lastly, this app only showed an improvement in adherence for 6 months; therefore, future studies are needed for long-term improvement.

### Conclusion

The Smart AF app improved medication adherence among elderly patients with AF. In the realm of medication management, an approach using an mHealth technology that emphasizes education, automatic reminder, and patient engagement may be helpful. The challenge that emerged, however, was the decline in the rate of persistent use of the app over time; therefore, continuous doctor-patient interaction via the app will be necessary in the future.

## Abbreviations

AEGEAN: Assessment of an Education and Guidance Programme for Eliquis Adherence in Non-Valvular Atrial Fibrillation
AF: atrial fibrillation
CHADS2: congestive heart failure, hypertension, age, diabetes, previous stroke, or transient ischemic attack
DOAC: direct oral anticoagulant
FACILITA: strategies for improving dabigatran adherence for stroke prevention in patients with non-valvular atrial fibrillation
HADS: Hospital Anxiety and Depression Scale
IMPACT-AF: Integrated Management Program Advancing Community Treatment of Atrial Fibrillation
JRISTA: the BMS/Pfizer Japan Thrombosis Investigator Initiated Research Program
mHealth: mobile health
MMAS-8: 8-item Morisky Medication Adherence Scale
OAC: oral anticoagulation
